# Human beta defensin levels and vaginal microbiome composition in post-menopausal women diagnosed with lichen sclerosus

**DOI:** 10.1038/s41598-021-94880-4

**Published:** 2021-08-06

**Authors:** Alexandra Brunner, Márta Medvecz, Nóra Makra, Miklós Sárdy, Kinga Komka, Máté Gugolya, Dóra Szabó, Márió Gajdács, Eszter Ostorházi

**Affiliations:** 1 Department of Dermatology, Venereology and Dermatooncology, Semmelweis University, Budapest, Hungary; 2 Institute of Medical Microbiology, Semmelweis University, Budapest, Hungary; 3 Department of Chemical and Environmental Process Engineering, Budapest University of Technology and Economics, Budapest, Hungary; 4 Department of Pharmacodynamics and Biopharmacy, Faculty of Pharmacy, University of Szeged, Szeged, Hungary

**Keywords:** Microbiology, Diseases

## Abstract

Human beta defensins (hBDs) may play an important role in the progression of lichen sclerosus (LS), due to their ability to induce excessive stimulation of extracellular matrix synthesis and fibroblast activation. The genetic ability of the individual to produce defensins, the presence of microbes influencing defensin production, and the sensitivity of microbes to defensins together regulate the formation of an ever-changing balance between defensin levels and microbiome composition. We investigated the potential differences in postmenopausal vaginal microbiome composition and vaginal hBD levels in LS patients compared to non-LS controls. LS patients exhibited significantly lower levels of hBD1 (*p* = 0.0003), and significantly higher levels of hBD2 (*p* = 0.0359) and hBD3 (*p* = 0.0002), compared to the control group. The microbiome of the LS patients was dominated by possibly harmful bacteria including *Lactobacillus iners, Streptococcus anginosus* or *Gardnerella vaginalis* known to initiate direct or indirect damage by increasing defensin level production. Our observations highlight that correcting the composition of the microbiome may be applicable in supplementary LS therapy by targeting the restoration of the beneficial flora that does not increase hBD2-3 production.

## Introduction

Lichen sclerosus (LS) is a chronic dermatosis of unknown origin, concentrated on the anogenital area compared to other cutaneous sites^1^. The overall prevalence of LS is estimated at 0.2%, and is observed in the male population less frequently than in the female population^2^. The genital form—which may present before the onset of puberty—is the most common in young girls, with atrophy of the skin and mucous membranes, in addition to bullous and hemorrhagic symptoms. As the chronic process of LS progresses, shrinkage of the connective tissue and scarring leads to pronounced narrowing of the vaginal opening with the affected surfaces being eroded and sensitive. Post-menopausal women are also commonly symptomatic. In fact, symptoms often worsen after the climacteric^3^.

The possible causes of the disease include genetic predisposition, chronic irritation, infection and autoimmunity^4^. Auto-antibodies targeting extracellular matrix 1 (ECM1) protein have been demonstrated in women with anogenital LS significantly more frequently (74%) compared with 7% in controls^5^. However, ECM1 autoreactivity might be involved in disease progression, rather than in the initiation of the condition. ECM1 autoreactivity occurs more likely in patients, whose symptoms persisted for longer than 1 year and/or in those with more extensive disease presentations. Antibodies develop in patients with autoimmune diathesis due to chronic irritation of the genital epithelium^6^. It has been proposed that some microbial “irritants” indirectly play a role in autoimmune processes and that the postmenopausal change of vaginal microbiome may be linked to the progression of LS. *Gardnerella vaginalis* as a participant in the dysbiotic or Bacterial Vaginosis (BV) associated vaginal microbiome plays not only an indirect role but also a direct role in inhibiting wound healing with its secreted soluble products^7^.

Human β defensins (hBDs) belong to the group of cysteine-rich short-chain natural antibacterial peptides. β-defensins are subdivided into further subgroups: hBD1 is produced in the kidney, in the epithelial cells of the respiratory tract and in the female genital tract constitutively, while hBD2 and hBD3 are inducible, expressed in inflammatory diseases of the skin. In addition to their known antibacterial activity, they contribute to immunomodulatory and chemotactic effects in inflammatory processes, infections and wound healing^8^. Lower relative mRNA expression of hBD1, but significantly higher hBD2 and hBD3 mRNA expression levels in LS patients, compared to healthy controls are observed^9^. Higher amounts of different hBDs in LS may change the appearance of the skin resembling pathological scarring, due to excessive stimulation of matrix synthesis and fibroblast activation. Pathogens of all sorts of infections induce production of ß defensins^10–12^. In turn, the increased levels of these peptides affects the composition of the surrounding bacterial flora due their selective antimicrobial activity^13,14^.

In the study of Glienwitz et al.^15^ two-thirds of postmenopausal healthy patients had a *Streptococcus* dominated microbiome, one-fifth of individuals had a *Gardnerella* dominated microbiome, while others belonged to *L. crispatus* or *L. iners* dominated clusters. Although the vaginal microbiome with the most optimal composition is dominated by *L. crispatus*, in many patients without clinical issues the proportion of other bacteria is higher. Therefore, if a pathological condition emerges, in a complex environment like the genital tract not only the bacterial composition has to be assessed, but a number of other factors.

The genetic ability of an individual to produce human defensins, the presence of microorganisms influencing defensin production in the surrounding environment of producer cells, and the sensitivity of microbes to defensins together regulate the formation of an ever-changing balance between defensin levels and microbiome composition. Menopause may be a time of reduced genital tract health, reflecting changes in the vaginal microbiome and mucosal environment.

In our current study, we aimed to investigate postmenopausal vaginal microbiome and associated defensin levels in LS and control patients.

## Results

Participants in both the LS (15) and control (8) groups were postmenopausal. All LS patients had a histologically confirmed illness for at least 9 years, and they all had subjective symptoms and objective signs at the time of the study (Suppl. Table 1–3). The assessment of symptom severity is summarized in Table 1. In LS patients hBD1 levels were significantly lower (median: 297 ng/mL) than in the CTL group (median: 975 ng/mL) (*p* = 0.0003), while hBD2 (LS median: 1110 pg/mL and CTL median: 614 pg/mL) (*p* = 0.0359) and hBD3 levels (LS median: 2998 ng/mL and CTL median: 994.5 ng/mL) (*p* = 0.0002) were significantly elevated in the LS group, measured in 10 mL cervicovaginal lavage. Based on subjective evaluation, the most severe symptoms were in patient LS7, and the mildest in patients LS6, LS10, LS11, LS14. Patient LS1 had the highest global objective score and patient LS7 had the lowest. Günthert^16^ severity score was the highest in patients LS1 and LS9, and the lowest in LS2, LS7, LS11 and LS12. Although there are discrepancies between subjective and objective severity assessments, none of the scores show a relationship between severity and the microbiome-determining dominant bacterial genus. No significant correlations, or any trends were found between symptom severity score values and hBD levels in any given LS patient.

**Table 1:**
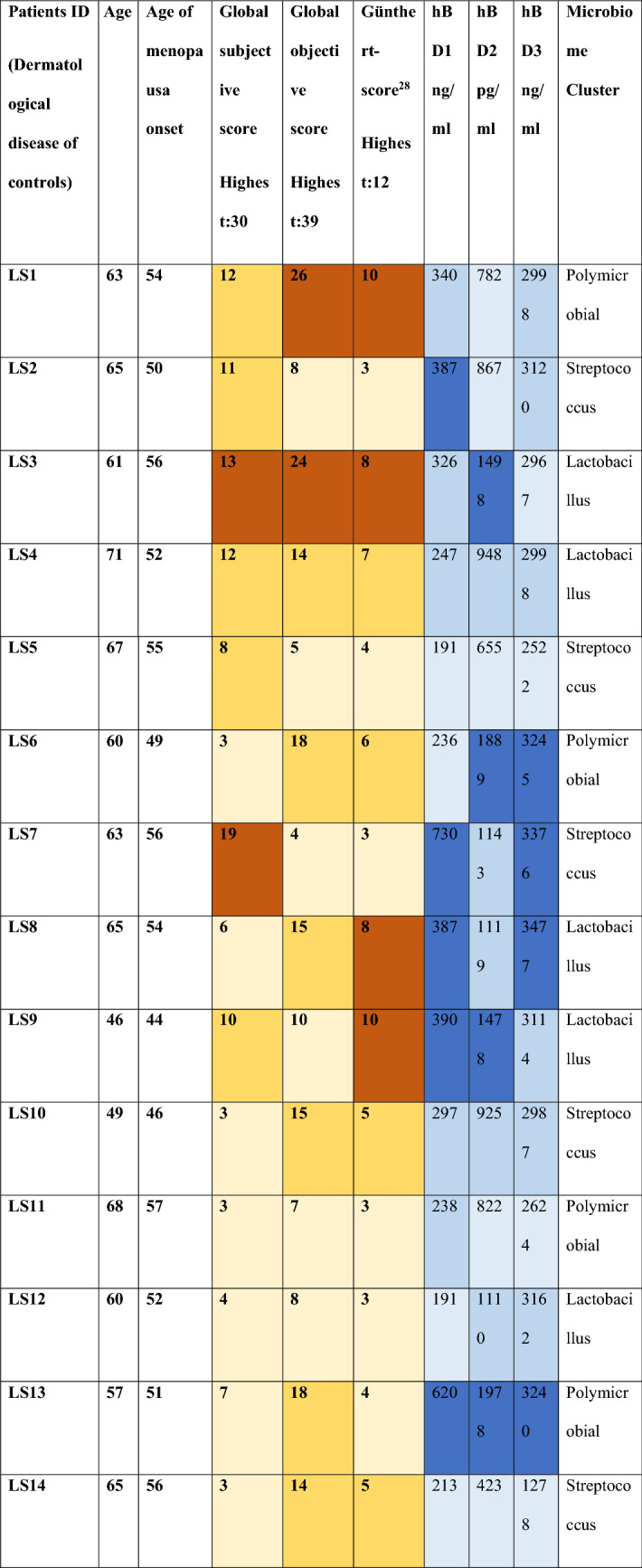

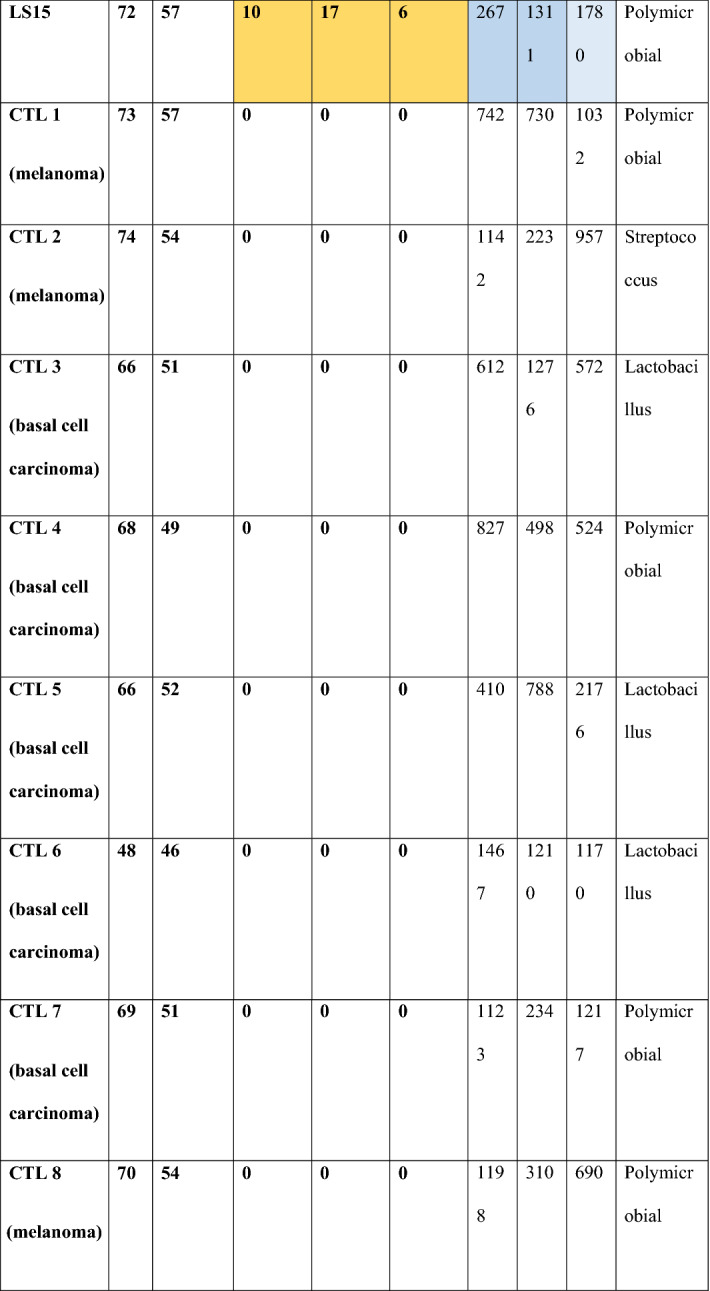
Individual patient-level data for patients in the LS and CTL groups, including dermatological diseases of controls, age, age of menopausa onset, global subjective score, global objective score, Günther score, vaginal defensin levels, and the genus that dominates the cluster. A darker color tone indicates that the score value (brown) or hBD level (blue) belongs to a higher quartile.

**Figure 1:**
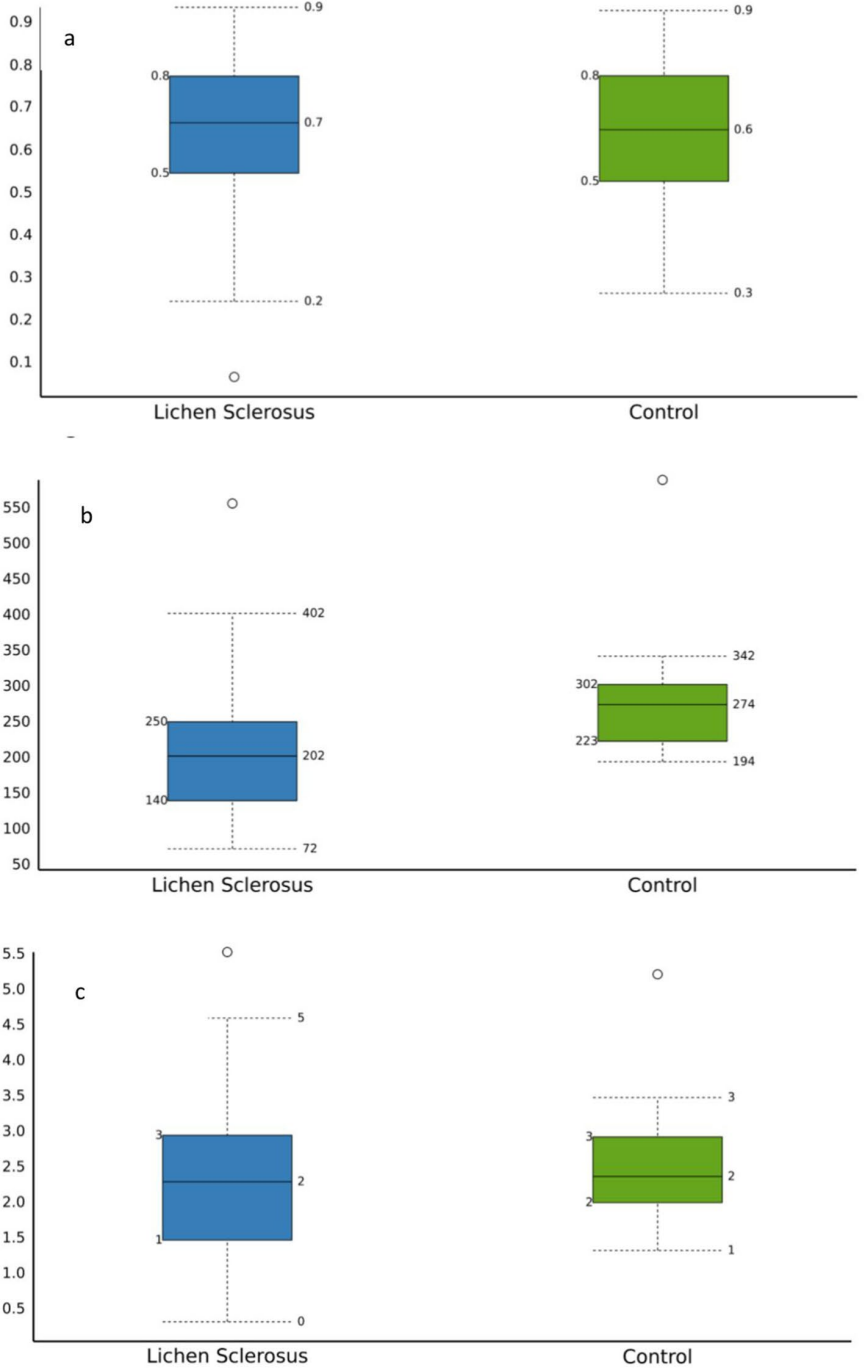
Microbial alpha diversity in LS and control patients. Wilcoxon rank sum testing found no significant difference by either method (**a)** Simpson, (**b)** Chao1, (**c)** Shannon alpha diversity analysis.

A total of 9.8 million valid sequences were obtained, resulting in 5.6 million high-quality reads; the median number of reads within one sample was 241,678 (IQR: 36,119). No statistical significant differences were found in microbial alpha diversity in the samples between LS and CTL patients by either metrics used to assess differences (Fig. 1a: Simpson, 1b: Chao1, 1c: Shannon alpha diversity analysis) with Wilcoxon rank sum testing at species level.

**Figure 2:**
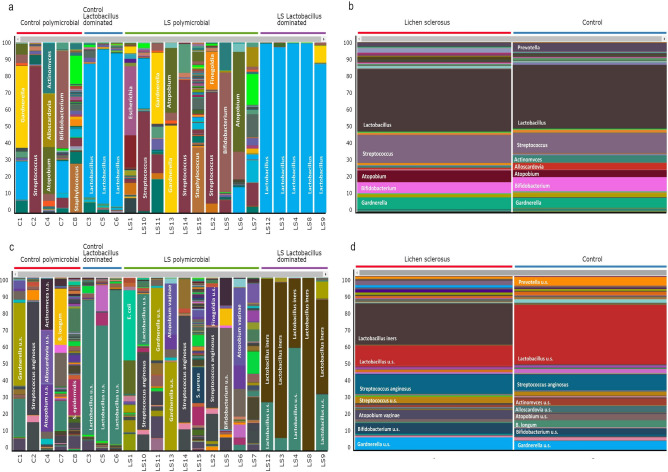
Vaginal microbiome composition of LS and control patients. (**a)** at genus level, one-third of the patients had a *Lactobacillus* dominated microbiome both in LS (5/15) and CTL (3/8) groups. (**b)** At genus level aggregated by cohorts: There were no significant differences among Streptococcus (*p* = 0.757) or Lactobacillus (*p* = 0.957) between the LS and Control group at genus level. **(c)** at species level, (**d)** at species level aggregated by cohorts Difference between the abundance was significant of *L. iners* (0,027), but no significant of *S. anginosus* species in the LS or Control group (*p* = 0,832).

Regardless of whether the patients belonged to the LS or the control group, they were equally distributed among the *Lactobacillus* or polymicrobial mainly *Streptococcus* or *Gardnerella-Atopobium*-dominated clusters. At genus level, one-third of the patients had a *Lactobacillus* dominated microbiome both in LS (5/15) and the control (3/8) groups (Fig. 2a). There is no significant difference in the genus dominance of the groups using the chi square test (*p* = 0.842). Aggregated by cohorts at genus level, the microbiome composition of LS cohort consisted of 35% *Lactobacillus* and 16% *Streptococcus*, while the control cohort contains 36% *Lactobacillus* and 12% *Streptococcus* (Fig. 2b). There were no significant differences among Streptococcus (*p* = 0.757) or Lactobacillus (*p* = 0.957) abundance between the LS and Control group at genus level. Moving on to the species-level analyses, a more striking difference was observed: among the Lactobacilli, *L. iners* species was present in an exceptionally high proportion in the LS group against the control group (*p* = 0,027) (Fig. 2c, d) (Suppl. Table 4). There was no significant difference between the abundance of *S. anginosus* species in the LS or control group (*p* = 0,832).

**Figure 3:**
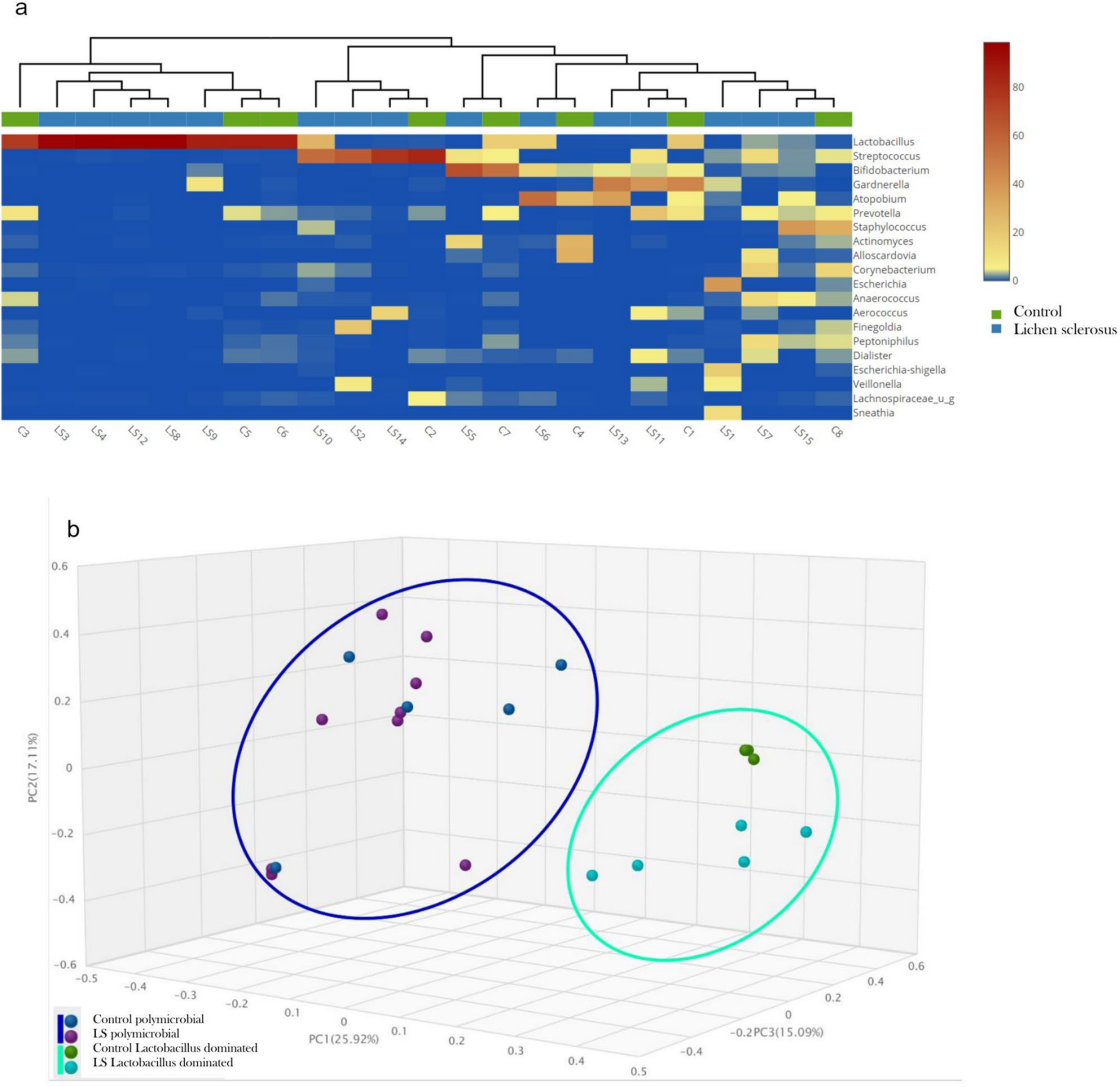
Polymicrobial and lactobacillus dominated clusters of LS and control samples on heatmap (**a**) and with Bray–Curtis principal coordinate analysis (PCoA) (**b**). Samples from two distinct clusters: cluster 1 contains samples with polymicrobial bacterial population; cluster 2 is dominated by members of Lactobacillus. Both clusters include LS and control patients. According to PERMANOVA analysis, significant difference among the LS and CTL group is not observed.

Figure 3a shows in Heatmap with a dendrogram annotation how the samples at genus level separated in two clusters regardless of whether they belonged to the control or LS group. Figure 3b Bray–Curtis Principal Coordinate Analysis (PCoA) showed also that the samples separated into two clusters, both the clusters contained both LS and control samples. Cluster 1 contained the samples characterized by a polymicrobial bacterial population, while Cluster 2 samples are dominated by *Lactobacillus*. According to PERMANOVA analysis, significant differences among the LS and control were not observed at species level. If the LS and control groups were divided into additional cohorts based on lactobacillus dominance or polymicrobial property, the ß diversity of only lactobacillus-dominant control and LS cohort differed significantly by PERMANOVA analysis. For a complete analysis please consult Table 2.

**Table 2:** PERMANOVA analysis data of the Bray–Curtis ß-diversity PCoA. The table lists for each cohort combination the number of included samples, the number of carries our permutations and normalized *p* value.

Cohorts	Sample size	Permutation	*p* value
Lichen sclerosus ↔ Control	23	999	0.117
Control polymicrobial ↔ Control Lactobacillus dominated	8	999	0.033
Control polymicrobial ↔ LS polymicrobial	15	999	0.979
Control polymicrobial ↔ LS Lactobacillus dominated	10	999	0.009
Control Lactobacillus dominated ↔ LS polymicrobial	13	999	0.009
Control Lactobacillus dominated ↔ LS Lactobacillus dominated	8	999	0.016
LS Lactobacillus dominated ↔ LS polymicrobial	15	999	0.002

**Figure 4:**
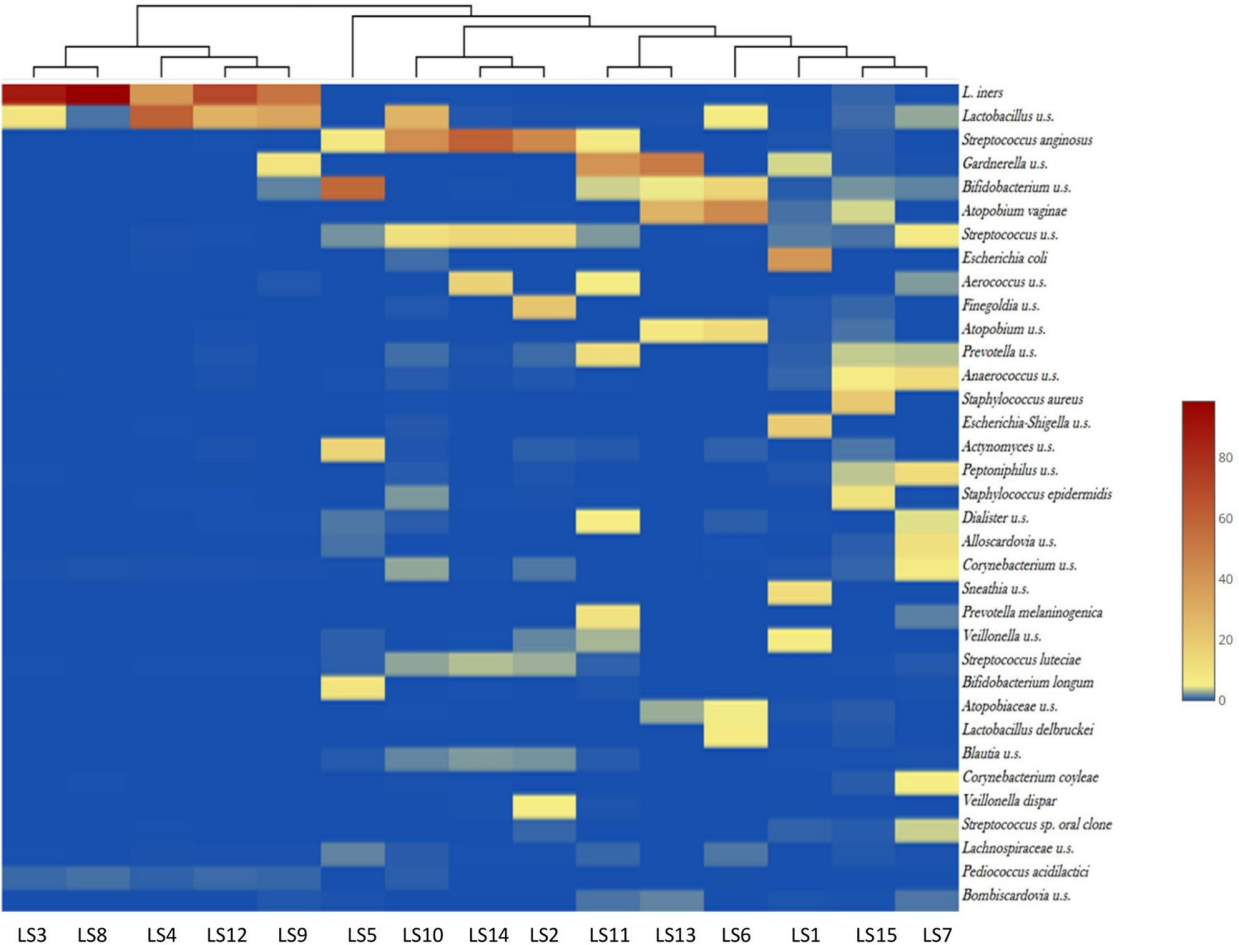
Heatmap visualization of the 35 most abundant taxa at species level among the LS patients. *L. iners* is associated mainly with *Lactobacillus u.s*. and *Pediococcus acidilactici. S. anginosus* had frequent coexistence only with other *Streptococcus sps*, or *Corynebacterium u.s*. In the further polymicrobial samples the most abundant species were *Gardnerella u.s., Bifidobacterium u.s*. and *Atopobium vaginae*.

Figure 4 shows a heatmap visualization of the 35 most abundant taxa at species-level among LS patients. In patients where *L. iners* was the most common species with a relative abundance between 68–96%—with the exception of *Lactobacillus u.s*. and *Pediococcus acidilactici*—other notable species were not detected in the vaginal microbiome. The only exception was the LS9 sample, where both *Gardnerella u.s*. and *Bifidobacterium u.s.* were associated with *L. iners*. *S. anginosus* was frequently co-existing almost excusively with other *Streptococcus sp.*, or *Corynebacterium u.s*. In the polymicrobial group, the most abundant species were *Gardnerella u.s., Bifidobacterium u.s*. and *Atopobium vaginae* frequently co-existing.

**Figure 5:**
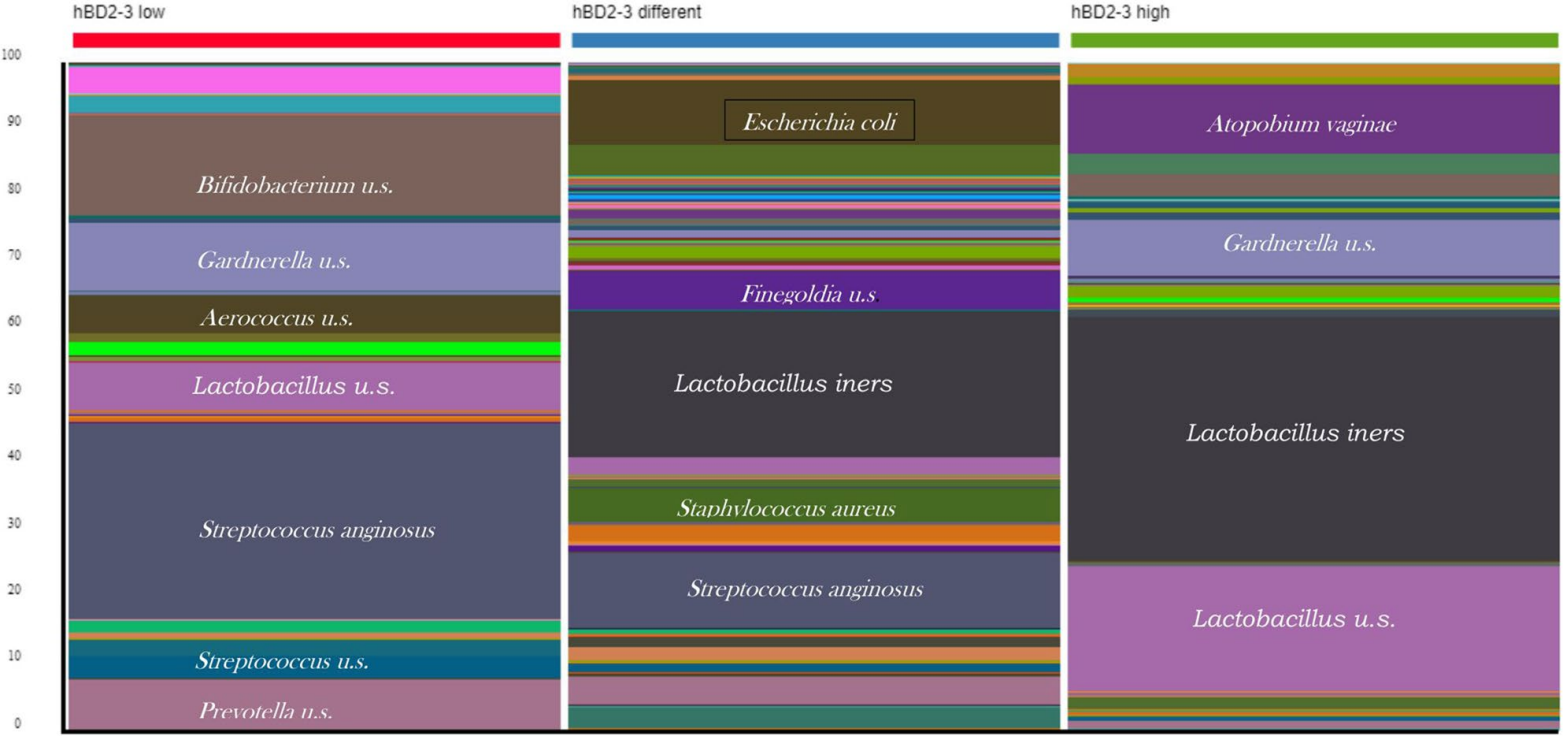
Vaginal microbial composition distribution according to the local levels of defensins. The prevalence of *L. iners* increases in parallel with the increase in hBD2-3 levels, while the prevalence of *S anginosus* decreases.

LS patients were divided into 3 distinct groups, based on the levels of hBD2, hBD3, and median LS defensin values. The first cohort includes patients whose hBD2 and hBD3 levels were lower than the median values (LS5, LS10, LS11, LS14). The second cohort contains LS patients whose hBD2 or hBD3 levels were higher than the median values (LS1, LS2, LS3, LS15), and patients in the third cohort had higher levels of both inducible hBDs than the median values in the LS group (LS4, LS6, LS7, LS8, LS9, LS12, LS13). Figure 5 shows that the amount of *L. iners* in the samples increased in parallel with hBD2 and hBD3 levels. However due to the high SD values, and low sample size significant differences were observed only between the lowest and highest hBD groups (First cohort ↔ second cohort: *p* = 0,387, second cohort ↔ third cohort: *p* = 0.592, first cohort ↔ third cohort: *p* = 0.046). Of note, the incidence of *Streptococcus anginosus* changed in an opposite direction to hBD2-3 levels, but the differences are not significant due to high SD. (First cohort ↔ second cohort: *p* = 0,331, second cohort ↔ third cohort: *p* = 0.385, first cohort ↔ third cohort: *p* = 0.109).

## Discussion

All patients in the LS group had a positive diagnosis of Lichen sclerosus for at least 9 years, but at the time of sampling they presented themselves for examination because their symptoms had worsened. The vaginal microbiome of healthy women during menopause can be different^15^ from the ideal microbiome dominated by *L. crispatus*, without any symptoms or disease. With increasing age, a number of individual factors and hormonal changes shape the microbiome that develops during menopause. LS is probably a multicausal disease in which individual genetic factors, microorganisms, and autoimmunity play roles in its formation and progression. There were patients in the healthy control group whose microbiome was *Streptococcus*-dominated or polymicrobial but did not have any pathological symptoms. Our working hypothesis is that potentially disease-causing bacteria, that form the microbiome of LS patients, are involved in the progression of this multicausal disease.

Each of the bacteria predominantly present in LS patients has virulence factors that can worsen the prognosis of the disease. Not all vaginal *Lactobacillus* species are equally beneficial to the host. *L. crispatus* is the optimal species associated with vaginal health, whereas *L. iners* may be associated with the development of pathological conditions^17^. The most important virulence factors of *L. iners* are inerolysin, and AB-1 adhesine^18^. Inerolysin is a pore-forming cholesterol dependent cytolysin toxin, which interacts with the CD59 human cell surface receptor, and at the end of a multi-step process it induces perforation of the cell membrane and ultimately cell death^19^. The AB-1 adhesine attaches to human fibronectin^18^. Fibronectin is one of the extracellular matrix components whose expression and distribution are altered in lichen sclerosus^20^. Further altered components are tenascin, fibrinogen, biglycan, versacin and ECM-1^21^. The alteration in these extracellular matrix components may be relevant to the initiation of scarring in LS and to the associated increased skin fragility^20^.

*S. anginosus* is a pathogenic species, the predominant microorganism in patients with aerobic vaginitis. Successful binding of these bacteria to extracellular matrix proteins, like fibronectin, fibrinogen and laminin plays an important role in their pathogenesis^22^. The *sag* haemolysin of *S. anginosus* has been described to initiate vaginal epithelial cell lysis^23^.

*Gardnerella vaginalis* and *Atopobium vaginae* are thought to be etiologic agents of bacterial vaginosis (BV). *G. vaginalis* is able to effectively displace lactobacilli and adhere to vaginal epithelial cells^24^, and has an increased propensity for biofilm formation^25^. Enzymes produced by *G. vaginalis*—vaginal sialidase or vaginolysin—promote the breakdown of the mucous layer and the vaginal epithelium^26^. Mature biofilm facilitates the adhesion of second colonizers, including *A. vaginae*^27^. *A. vaginae* induces a broad range of pro-inflammatory cytokines, chemokines, and antimicrobial peptides, including IL-1β, IL-6, IL-8, MIP-3α, hBD-2 and TNFα^17^. Some of the bacteria in the vaginal microbiome are known to play a role in enhancing antimicrobial peptide production: hBD2 levels are most strongly elevated in the presence of *A. vaginae, P. bivia* and *L. iners* without any effect on hBD1 production^17^.

Gambichler^9^ and co-workers measured significantly lower hBD1 mRNA expression, and higher hBD2 and hBD3 mRNA expressions in LS patients than in controls. In our study LS patients had significantly lower levels of hBD1 (*p* = 0.0003), and significantly higher levels of hBD2 (*p* = 0.0359) and hBD3 (*p* = 0.0002), compared to the control group. Also psoriasin, LL-37 and RNAse 7 were analysed in the above mentioned study, and measured a higher level of constitutively expressed psoriasin in LS patients but no differences between the levels of inducible LL-37 and RNAse 7 in LS patients and control groups. Further studies are needed to characterize the factors influencing the prevalence of bacterial species in a complex environment such as the vagina.

Increased levels of hBD2 and hBD3 levels were correlated with higher amounts of *Lactobacillus sp.* in the vaginal microbiome^28^. During our study, the detected concentration of defensins overall was about 2 µg/mL in the 10 mL of lavage fluid; however, this may reflect a considerably higher concentration directly on the mucosal surface. It would be reasonable to speculate that the survival of different *Lactobacillus* species and other bacteria is largely affected by these amounts of different defensins. Antimicrobial peptide (AMP) susceptibility and the capability of different bacteria to induce the production of AMPs may explain the difference in the levels of defensins in LS patients and controls, and can affect the composition of the corresponding various microbiomes. In both the control and LS patient groups, the presence of *L. iners* in the microbiome was only observed at low hBD1 levels. Further studies are needed to investigate whether low hBD1 levels are a prerequisite for *L. iners* to exist in the vaginal microbiota. The low level of hBD1 in LS patients may explain the differences in *Lactobacillus* species present in patients, compared to controls. Based on our results, it appears that in LS patients, characterized by low hBD1 levels, a series of bacterial species are present, as opposed to the healthy flora dominated by only *L. crispatus*. Consequently, as hBD2 and hBD3 levels are increased, the total amounts of *S. anginosus* decreased and the presence of *L. iners* is increased.

Limitations of this study are the small number of patients, the exclusive use of the 16S rRNA sequencing method, that provides species-level identification in only a few cases and the lack of proteomic analysis. This latter would highlight the importance and relationship of additional antibacterial peptides and bacterial products in patients diagnosed with LS.

In summary, we observed differences in both defensin levels and the microbial composition in the samples obtained from LS patients compared to the samples from non-LS patients. Although the differences were clearly observable, additional studies are warranted to explore the cause-and-effect relationship between defensin levels and the presence/absence of various microorganisms (e.g., *L. iners*). Consideration should be given to supplement LS therapy with *Lactobacillus*-containing probiotics, or to restore the beneficial flora that does not induce the increase in hBD2-3 production, in order to improve the quality of life in patients affected by LS. It would be worthwhile to investigate whether higher levels of hBD1 are required for the colonization of beneficial lactoflora.

## Methods

### Subject Recruitment

Twenty-three (n = 23) postmenopausal women were recruited at the Department of Dermatology, Venerology and Dermatooncology of Semmelweis University between March 2018 and September 2018. Sample collection began following approval from the Ethics Committee of the Semmelweis University (SE TUKEB: 275/2017). All procedures performed in the studies involving human participants were in accordance with the ethical standards of the institutional and/or national research committee and with the 1964 Helsinki Declaration and its later amendments or comparable ethical standards, and participants gave written informed consent for sample collection and analysis for research purposes.

The participants included women in an LS and a control (CTL) group. The LS group included n = 15 women, diagnosed with LS based on histological findings. Members of the LS group suffered from different active symptoms or refused them. The CTL group had n = 8 individuals, who were patients of the Department with other dermatological diseases (melanoma or basal cell carcinoma), who voluntarily agreed to have their vaginal secretions examined. Only individuals (patients and controls), that were not taking antibiotics or immunosuppressive medications for any reason in the 3 months prior to sample collection were included in our study. Exclusion criteria for both groups were: positive history of sexually transmitted or recent genital infections, use of lactobacillus-containing suppository or gynecological intervention in the last 3 months. In all cases, the physical examination was preceded by the completion of a questionnaire on previous illnesses, their treatment and current complaints. The LS score classification was based on a subjective scoring of relevant symptoms, an objective score and the Günthert classification^16^. Subjective scores for pruritus, burning sensation and dyspareunia were quantified by interview, using a visual analogue scale (VAS, which included a numeric rating scale 0–10). A global subjective score (GSS) was obtained by summing the scores of each symptom parameter (highest GSS = 30.) The following objective parameters were scored to evaluate clinical feature of the patients: (1) leukoderma (2) sclerosis (3) atrophy (4) fine wrinkling (5) lichenification (6) hyperkeratosis (7) erosion (8) oedema (9) erythema (10) purpuric lesions (11) itching-related excoriations (12) unilateral labial adhesion (13) bilateral labial adhesion. Each sign was scored using the following 4-point scale: 0 = absence, 1 = mild, 2 = moderate, 3 = severe. A global objective score (GOS) was obtained by summing the scores of each clinical parameter (highest GOS = 39). The Günthert score was calculated by measuring (1) erosion (2) hyperkeratosis (3) fissures (4) agglutination (5) stenosis (6) atrophy (0 = absence, 1 = mild 2 = severe; global score maximum: (12). The characteristics of the study participants are presented in Table 1. and in Supplementary Table 1, 2 and 3.

### Sample collection

For vaginal microbiome analysis, swab samples were collected by using the Puritan UniTranz-RT transport system (Puritan Medical Products, Guilford, USA). Cervicovaginal lavage was collected by washing the cervix and vagina with 10 mL of normal saline and supernatants were aliquotted and stored at − 80 °C for determination of defensin levels^29^.

### DNA isolation

DNA isolation was performed according to manufacturer’s protocol by the ZymoBIOMICS DNA Miniprep Kit (Zymo Research Corp.,Irvine, USA). Concentration of genomic DNA was measured using a Qubit2.0 Fluorometer with Qubit dsDNA HS Assay Kit (Thermo Fisher Scientific, Waltham, MA, USA). Bacterial DNA was amplified with tagged primers covering the V3-V4 region of bacterial 16S rRNA gene. PCR and DNA purification were performed according to Illumina’s protocol. PCR product libraries were assessed using DNA 1000 Kit with Agilent 2100 Bioanalyzer (Agilent Technologies, Waldbronn, Germany). Equimolar concentrations of libraries were pooled and sequenced on an Illumina MiSeq platform (Illumina, San Diego, CA, USA), using MiSeq Reagent Kit v3 (600 cycles PE). In order to evaluate contribution of extraneous DNA from reagents, extraction negative controls and PCR negative controls were included in every run. To ensure reproducibility, each sample was independently extracted and sequenced twice. Isolated DNA samples were placed at − 80 °C until PCR amplification. Raw sequencing data were retrieved from the Illumina BaseSpace and uploaded to CosmosId Bioinformatics Platform for evaluation (CosmosID Metagenomics Cloud, app.cosmosid.com, CosmosID Inc., www.cosmosid.com).

### hBD ELISA

The following ELISA kits were used for quantitative measurement of human ß defensins, according to manufacturer instructions: SEB373Hu for hBD1, SEA072Hu for hBD2 and SEE132Hu for hBD3 (Cloud-Clone Corp. Houston, USA). All diluted standards, samples and blank wells were measured in duplicates.

### Statistical analysis

The levels of statistical significance for the difference between vaginal defensin levels , and bacterial taxa abundances—not normally distributed variables—measured in the LS and CTL groups was calculated by Mann–Whitney U test. The difference in the incidence of taxa was assessed by chi-square test. Statistical significance between cohorts were implemented using Wilcoxon rank sum testing for microbiome alpha diversity (Chao1, Simpson, Shannon indexes) and PERMANOVA analysis for Bray–Curtis PCoA beta diversity using the statistical analysis support application of CosmosID (CosmosID Metagenomics Cloud, app.cosmosid.com, CosmosID Inc., www.cosmosid.com).

## Supplementary Information

Supplementary Information 1.

Supplementary Information 2.

Supplementary Information 3.

Supplementary Information 4.

## Data Availability

The datasets generated during the current study are available in the Short Read Archive (SRA) of National Center for Biotechnology Information under accession number: PRJNA693292, http://www.ncbi.nlm.nih.gov/bioproject/693292.
