# Supplementary material for: Human beta defensin levels and vaginal microbiome composition in post-menopausal women diagnosed with lichen sclerosus
Source: Sci Rep. 2021 Aug 6;11:15999. doi: 10.1038/s41598-021-94880-4 (PMC8346569; doi:10.1038/s41598-021-94880-4)
Supplement: Supplementary file 1 — Supplementary Information 1. [file 41598_2021_94880_MOESM1_ESM.docx]

**Supplementary Table 1:** Individual patient-level data for patients in the LS and CTL groups, including data for Subjective Severity Score of Lichen Sclerosus

| Patients Id | Pruritus | Burning sensation | Dyspareunia | Subjective Score |
| --- | --- | --- | --- | --- |
| LS1 | 2 | 0 | 10 | 12 |
| LS2 | 6 | 5 | 0 | 11 |
| LS3 | 5 | 5 | 3 | 13 |
| LS4 | 0 | 9 | 3 | 12 |
| LS5 | 0 | 0 | 8 | 8 |
| LS6 | 0 | 0 | 3 | 3 |
| LS7 | 8 | 8 | 3 | 19 |
| LS8 | 3 | 0 | 3 | 6 |
| LS9 | 3 | 3 | 4 | 10 |
| LS10 | 0 | 0 | 3 | 3 |
| LS11 | 0 | 0 | 3 | 3 |
| LS12 | 1 | 1 | 3 | 4 |
| LS13 | 4 | 0 | 3 | 7 |
| LS14 | 0 | 0 | 3 | 3 |
| LS15 | 0 | 0 | 10 | 10 |
| CTL1 | 0 | 0 | 0 | 0 |
| CTL2 | 0 | 0 | 0 | 0 |
| CTL3 | 0 | 0 | 0 | 0 |
| CTL4 | 0 | 0 | 0 | 0 |
| CTL5 | 0 | 0 | 0 | 0 |
| CTL6 | 0 | 0 | 0 | 0 |
| CTL7 | 0 | 0 | 0 | 0 |
| CTL8 | 0 | 0 | 0 | 0 |
