# Supplementary material for: Human beta defensin levels and vaginal microbiome composition in post-menopausal women diagnosed with lichen sclerosus
Source: Sci Rep. 2021 Aug 6;11:15999. doi: 10.1038/s41598-021-94880-4 (PMC8346569; doi:10.1038/s41598-021-94880-4)
Supplement: Supplementary file 2 — Supplementary Information 2. [file 41598_2021_94880_MOESM2_ESM.docx]

**Supplementary Table 2:** Individual patient-level data for patients in the LS and CTL groups, including data for Objective Severity Score of Lichen Sclerosus
