# Supplementary material for: Human beta defensin levels and vaginal microbiome composition in post-menopausal women diagnosed with lichen sclerosus
Source: Sci Rep. 2021 Aug 6;11:15999. doi: 10.1038/s41598-021-94880-4 (PMC8346569; doi:10.1038/s41598-021-94880-4)
Supplement: Supplementary file 3 — Supplementary Information 3. [file 41598_2021_94880_MOESM3_ESM.docx]

**Supplementary Table 3:** Individual patient-level data for patients in the LS and CTL groups, including data for Günthert Severity Score of Lichen Sclerosus

| **Patients Id** | **Erosion** | **Hyperkeratosis** | **Fissures** | **Agglutination** | **Stenosis** | **Atrophy** | **Günther Score** |
| --- | --- | --- | --- | --- | --- | --- | --- |
| **LS1** | **2** | **2** | **2** | **1** | **1** | **2** | **10** |
| **LS2** | **0** | **0** | **0** | **2** | **0** | **1** | **3** |
| **LS3** | **2** | **1** | **0** | **2** | **1** | **2** | **8** |
| **LS4** | **2** | **1** | **1** | **1** | **1** | **1** | **7** |
| **LS5** | **1** | **1** | **1** | **0** | **0** | **1** | **4** |
| **LS6** | **2** | **0** | **1** | **0** | **1** | **2** | **6** |
| **LS7** | **0** | **0** | **1** | **0** | **1** | **1** | **3** |
| **LS8** | **1** | **0** | **1** | **2** | **2** | **2** | **8** |
| **LS9** | **2** | **2** | **0** | **2** | **2** | **2** | **10** |
| **LS10** | **1** | **0** | **1** | **0** | **1** | **2** | **5** |
| **LS11** | **0** | **0** | **0** | **1** | **1** | **1** | **3** |
| **LS12** | **0** | **0** | **0** | **1** | **1** | **1** | **3** |
| **LS13** | **0** | **1** | **0** | **0** | **1** | **2** | **4** |
| **LS14** | **0** | **0** | **0** | **2** | **1** | **2** | **5** |
| **LS15** | **0** | **0** | **0** | **2** | **2** | **2** | **6** |
| **CTL1** | **0** | **0** | **0** | **0** | **0** | **0** | **0** |
| **CTL2** | **0** | **0** | **0** | **0** | **0** | **0** | **0** |
| **CTL3** | **0** | **0** | **0** | **0** | **0** | **0** | **0** |
| **CTL4** | **0** | **0** | **0** | **0** | **0** | **0** | **0** |
| **CTL5** | **0** | **0** | **0** | **0** | **0** | **0** | **0** |
| **CTL6** | **0** | **0** | **0** | **0** | **0** | **0** | **0** |
| **CTL7** | **0** | **0** | **0** | **0** | **0** | **0** | **0** |
| **CTL8** | **0** | **0** | **0** | **0** | **0** | **0** | **0** |
