# Supplementary material for: Human beta defensin levels and vaginal microbiome composition in post-menopausal women diagnosed with lichen sclerosus
Source: Sci Rep. 2021 Aug 6;11:15999. doi: 10.1038/s41598-021-94880-4 (PMC8346569; doi:10.1038/s41598-021-94880-4)
Supplement: Supplementary file 4 — Supplementary Information 4. [file 41598_2021_94880_MOESM4_ESM.docx]

**Supplementary Table 4 Data of statistical analysis of taxa abundance between the LS and Control groups.** The levels of statistical significance for the difference between taxa abundances – not normally distributed variables – measured in the LS and CTL groups was calculated by Mann-Whitney U test

| **Group/Taxa** | **Median** | **SD** | **p value** |
| --- | --- | --- | --- |
| **LS/Streptococcus genus** | **2.04** | **25.72** |  |
| **Control/Streptococcus genus** | **0.14** | **27.31** |  |
| **LS/Streptococcus genus** ↔**Control/Streptococcus genus** |  |  | **0.757** |
| **LS/Lactobacillus genus** | **2.72** | **43.24** |  |
| **Control/Lactobacillus genus** | **19.76** | **37.19** |  |
| **LS/Lactobacillus genus** ↔**Control/Lactobacillus genus** |  |  | **0.957** |
| **LS/L. iners** | **38.31** | **34.51** |  |
| **Control/ L. iners** | **0.03** | **0.03** |  |
| **LS/L. iners** ↔**Control/ L. iners** |  |  | **0.027** |
| **LS/ S. anginosus** | **0.03** | **19.03** |  |
| **Control/ S. anginosus** | **0.04** | **20.51** |  |
| **LS/ S. anginosus** ↔**Control/ S. anginosus** |  |  | **0.833** |
